# Altered Purinergic Receptor Sensitivity in Type 2 Diabetes-Associated Endothelial Dysfunction and Up_4_A-Mediated Vascular Contraction

**DOI:** 10.3390/ijms19123942

**Published:** 2018-12-07

**Authors:** Ali Mahdi, Tong Jiao, Yahor Tratsiakovich, Jiangning Yang, Claes-Göran Östenson, John Pernow, Zhichao Zhou

**Affiliations:** 1 Unit of Cardiology, Department of Medicine, Karolinska Institutet, Karolinska University Hospital, Stockholm 17176, Sweden; ali.mahdi@ki.se (A.M.); tong.jiao@ki.se (T.J.); yahor.tratsiakovich@ki.se (Y.T.); jiangning.yang@ki.se (J.Y.); john.pernow@ki.se (J.P.); 2 Endocrinology and Diabetology, Department of Molecular Medicine and Surgery, Karolinska Institutet, Karolinska University Hospital, Stockholm 17176, Sweden; Claes-Goran.Ostenson@ki.se

**Keywords:** purinergic receptors, endothelial dysfunction, Up_4_A, vascular contraction, diabetes

## Abstract

Purinergic signaling may be altered in diabetes accounting for endothelial dysfunction. Uridine adenosine tetraphosphate (Up_4_A), a novel dinucleotide substance, regulates vascular function via both purinergic P1 and P2 receptors (PR). Up_4_A enhances vascular contraction in isolated arteries of diabetic rats likely through P2R. However, the precise involvement of PRs in endothelial dysfunction and the vasoconstrictor response to Up_4_A in diabetes has not been fully elucidated. We tested whether inhibition of PRs improved endothelial function and attenuated Up_4_A-mediated vascular contraction using both aortas and mesenteric arteries of type 2 diabetic (T2D) Goto Kakizaki (GK) rats vs. control Wistar (WT) rats. Endothelium-dependent (EDR) but not endothelium-independent relaxation was significantly impaired in both aortas and mesenteric arteries from GK vs. WT rats. Non-selective inhibition of P1R or P2R significantly improved EDR in aortas but not mesenteric arteries from GK rats. Inhibition of A1R, P2X_7_R, or P2Y_6_R significantly improved EDR in aortas. Vasoconstrictor response to Up_4_A was enhanced in aortas but not mesenteric arteries of GK vs. WT rats via involvement of A1R and P2X_7_R but not P2Y_6_R. Depletion of major endothelial component nitric oxide enhanced Up_4_A-induced aortic contraction to a similar extent between WT and GK rats. No significant differences in protein levels of A1R, P2X_7_R, and P2Y_6_R in aortas from GK and WT rats were observed. These data suggest that altered PR sensitivity accounts for endothelial dysfunction in aortas in diabetes. Modulating PRs may represent a potential therapy for improving endothelial function.

## 1. Introduction

Type 2 diabetes (T2D) is an important risk factor for the development of cardiovascular disease including atherosclerosis and ischemic heart disease. Endothelial dysfunction is an early manifestation of the disease progression and plays a major role in the etiology of diabetes-induced macrovascular and microvascular complications [1,2]. The underlying cause of endothelial dysfunction is multifactorial and complex, but some of the key mechanisms include imbalance between endothelium-derived vasodilators such as nitric oxide (NO) and adenosine triphosphate (ATP), and vasoconstrictors such as endothelin, reactive oxygen species, and ATP, as well as receptor-mediated signaling activated by certain endothelium-derived factors, e.g., ATP-activated purinergic signaling [3,4].

Activation of purinergic receptors (PRs) by various extracellular nucleotides and nucleosides play a pivotal role in the control of vascular function. The purinergic receptor family consists of P1R and P2R based on their molecular structures and pharmacological moieties [5,6]. P1R, also named adenosine receptors, are divided into four subtypes: A1R, A_2A_R, A_2B_R, and A3R. P2R can be further divided into P2XR and P2YR [4]. To date, seven P2XRs and eight P2YRs have been recognized [4]. Of importance, purinergic signaling has been observed to be altered in both experimental animals and humans with T2D and such alteration may account for the development of endothelial dysfunction [7,8,9,10]. Indeed, ATP- and UTP-induced vascular contraction in mesenteric arteries was increased in rats with T2D, an effect that was attenuated by non-selective P2R inhibition [11]. In addition, the vasodilator response elicited by ATP was decreased in mesenteric arteries from rats with diabetes [12]. Similarly, the vasodilation to ATP, UTP, and adenosine was impaired in femoral arteries of patients with T2D [13]. However, due to a lack of specific antagonists for the most of PRs, the precise role of PRs in control of vascular function, in particular the contribution of those PRs to the development of endothelial dysfunction in T2D, remains to be determined.

A novel dinucleotide, uridine adenosine tetraphosphate (Up_4_A), was initially identified as an endothelium-derived vasoconstrictor, exerting its vasoconstrictor influence via both P1R and P2R in various vascular beds [14,15,16,17,18,19]. The vasoconstriction was shown to involve the generation of thromboxane (TxA2) [16] and reactive oxygen species (ROS) [18]. Of note, Up_4_A-induced contraction mediated by P2R was increased in renal arteries from type 2 diabetic Goto-Kakizaki (GK) rats [10]. Up_4_A-induced renal contraction was increased in Otsuka Long-Evans Tokushima Fatty (OLETF) T2D rats with age and duration of diabetes [9]. Our recent studies further revealed an altered purinergic signaling in response to Up_4_A in coronary microcirculation from diabetic swine [8]. Collectively, these observations support the notion that vascular purinergic singling is altered in diabetes.

The aim of our study was therefore to investigate the role of PRs in the regulation of endothelial function. We evaluated endothelial function based on acetylcholine (ACh)-induced endothelium-dependent relaxation (EDR) and used Up_4_A as pharmacological stimulator for purinergic activation in both conduit and resistance arteries (aortas and mesenteric arteries) from GK rats. Meanwhile, we used both the non-selective P1R and P2R antagonists as well as the specific antagonists available against A1R, P2X_7_R, and P2Y_6_R to identify the putative PRs that accounts for endothelial dysfunction in T2D.

## 2. Results

### 2.1. Characterization of Endothelial Function in Diabetic Rats

At the time of the experiment, GK rats had higher blood glucose compared to Wistar (WT) rats (10.8 ± 0.6 mmol/L in GK vs. 4.4 ± 0.2 mmol/L in WT, *p* < 0.001), but lower body weight (356 ± 5 g in GK vs. 481 ± 11 g in WT, *p* < 0.001).

**Figure 1:**
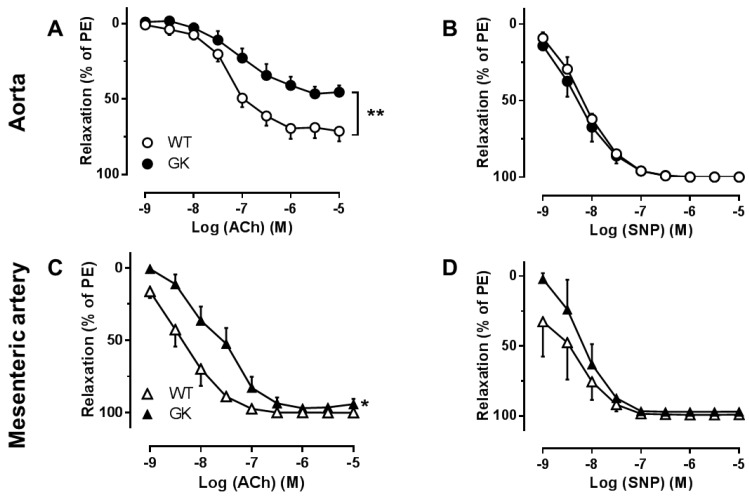
Concentration-response curve for acetylcholine (ACh) (**A**, *n* = 10–18), representing endothelium-dependent relaxation (EDR) or sodium nitroprusside (SNP), representing endothelium-independent relaxation (EIDR) (**B**, *n* = 3–4) in aortas isolated from Wistar (WT) and Goto-Kakizaki (GK) rats. EDR (**C**, *n* = 8–10) as well as EIR (**D**, *n* = 3) were also evaluated in mesenteric arteries from WT and GK rats. Data are presented as mean ± SEM as percentage relaxation of PE. * *p* < 0.05, ** *p* < 0.01, calculated with two-way ANOVA.

To determine endothelial function in both conduit and resistant arteries, ACh-induced EDR and sodium nitroprusside (SNP)-induced endothelium-independent relaxations (EIR) were conducted in aortas and mesenteric arteries preconstricted with phenylephrine (PE) from WT and GK rats. EDR (Figure 1A,C) but not EIR (Figure 1B,D) was significantly impaired in both aortas and mesenteric arteries isolated from GK rats as compared to WT rats (−logEC50: 8.4 ± 2.5 in WT mesenteric arteries; 7.7 ± 3.2 in GK mesenteric arteries, *p* < 0.05), indicating endothelial dysfunction in GK rats.

### 2.2. Effects of the Non-Specific P1R and P2R Antagonists on Endothelial Function in Aortas and Mesenteric Arteries

**Figure 2:**
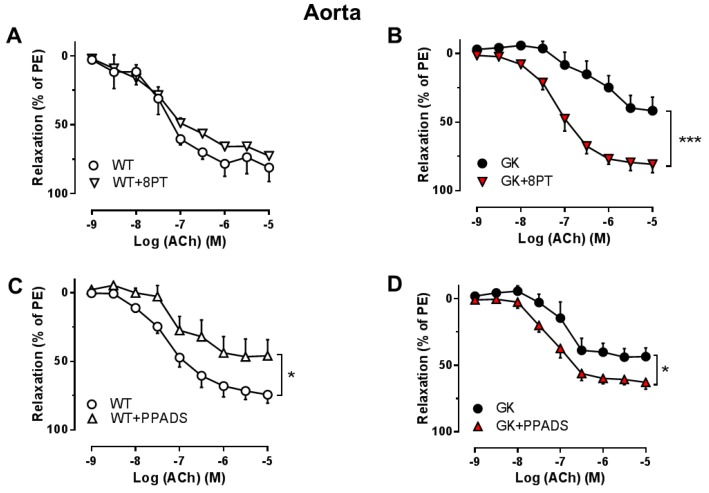
Effects of the P1R and P2R antagonists on EDR in aortas from WT and GK rats. Concentration-response curves for acetylcholine (ACh) in aortas preconstricted with PE in the absence and presence of the non-selective P1R antagonist (8PT, 10^−5^ M) from WT (**A**, *n* = 3) or GK (**B**, *n* = 6). Concentration-response curves for ACh in aortas preconstricted with PE in the absence and presence of the non-selective P2R antagonist (PPADS, 10^−5^ M) from WT (**C**, *n* = 5) or GK (**D**, *n* = 5). Data are presented as mean ± SEM as percentage relaxation of PE. * *p* < 0.05, *** *p* < 0.001, calculated with two-way ANOVA.

**Figure 3:**
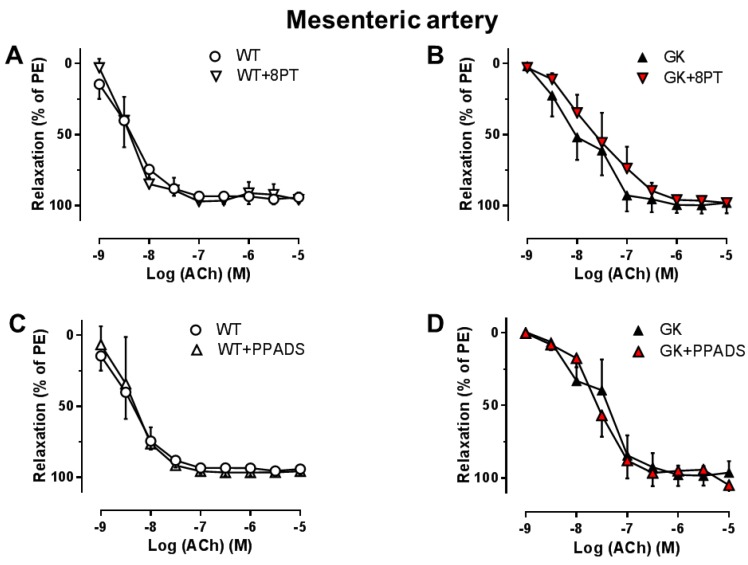
Effects of P1R and P2R antagonists on EDR in mesenteric arteries from WT and GK rats. Concentration-response curves for acetylcholine (ACh) in mesenteric arteries preconstricted with PE in the absence and presence of the non-selective P1R antagonist (8PT, 10^−5^ M) from WT (**A**, *n* = 3) or GK (**B**, *n* = 4). Concentration-response curves for ACh in mesenteric arteries preconstricted with PE in the absence and presence of the non-selective P2R antagonist (PPADS, 10^−5^ M) from WT (**C**, *n* = 3) or GK (**D**, *n* = 4). Data are presented as mean ± SEM as percentage relaxation of PE. No significant differences were detected with two-way ANOVA.

We investigated the effect of non-selective P1R and P2R inhibition on EDR in aortas and mesenteric arteries isolated from WT and GK rats. The non-selective P1R antagonist 8PT markedly improved EDR in GK aortas (Figure 2B), but had no effect on EDR in WT aortas (Figure 2A). Moreover, the non-selective P2R antagonist PPADS improved EDR in GK aortas (Figure 2D) but impaired EDR in WT aortas (Figure 2C). In contrast, neither 8PT (Figure 3A,B) nor PPADS (Figure 3C,D) affected EDR in mesenteric arteries from WT and GK rats. These observations indicate that involvement of PRs is altered contributing to endothelial dysfunction in conduit, but unlikely in resistance arteries in T2D.

### 2.3. Effects of the Specific Antagonists for A1R, P2X_7_R, and P2Y_6_R on Endothelial Function in Aortas

**Figure 4:**
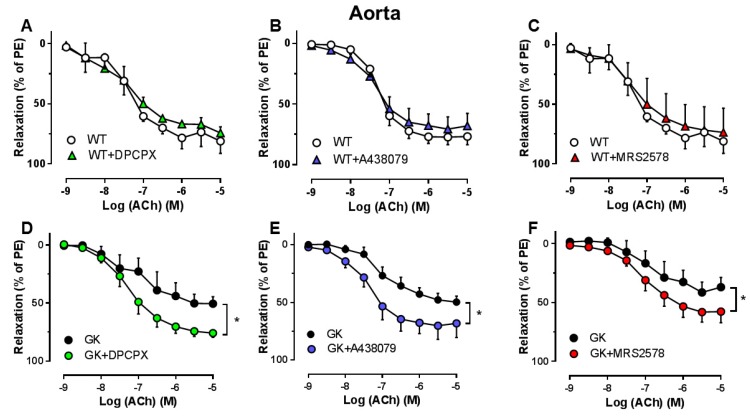
Effects of specific antagonism for A1R, P2X_7_R, and P2Y_6_R on EDR in aortas. Concentration–response curves for acetylcholine (ACh) in aortas preconstricted with PE in the absence and presence of the A1R antagonist (DPCPX, 10^−8^ M) (**A**, WT *n* = 3; D, GK *n* = 7), the P2X_7_R antagonist (A438079, 10^−5^ M) (**B**, WT *n* = 4; E, GK *n* = 10), and the P2Y_6_R antagonist (MRS2578, 10^−5^ M) (**C**, WT *n* = 3; F, GK *n* = 8). Data are presented as mean ± SEM as percentage relaxation of PE. * *p* < 0.05 calculated with two-way ANOVA.

Since both P1 and P2 inhibition affected endothelial function in aortas, but not mesenteric arteries, we further investigated involvement of specific PRs in endothelial function in aortas. A1R inhibition with DPCPX, P2X_7_R inhibition with A438079, and P2Y_6_R inhibition with MRS2578 significantly improved EDR in aortas from GK rats (Figure 4D–F), but had no effect on EDR in aortas from WT rats (Figure 4A–C).

### 2.4. Effects of Up_4_A on Vascular Function in Aortas and Mesenteric Arteries

**Figure 5:**
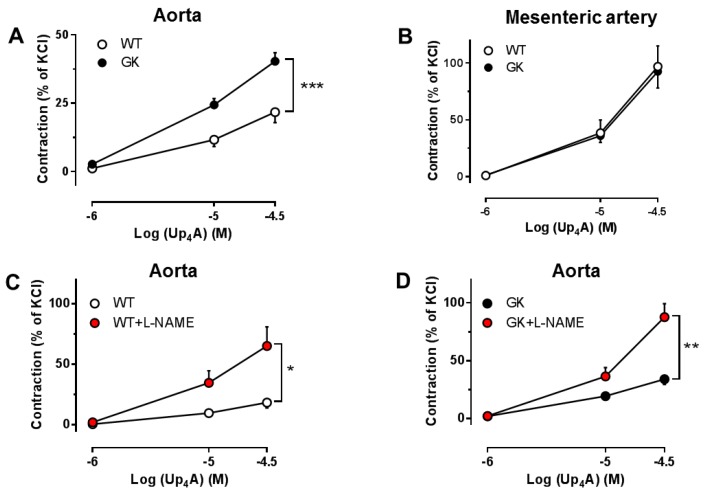
Vasoconstrictor response to Up_4_A at baseline in arteries from WT and GK rats. Comparison of concentration response curves to cumulative application of Up_4_A in aortas (**A**, WT *n* = 18, GK *n* = 32) or mesenteric arteries (**B**, WT *n* = 7, GK *n* = 8) between WT and GK rats. Effects of nitric oxide synthase inhibitor *L*-NAME on Up_4_A-induced contraction in aortas of WT and GK rats (**C**, WT *n* = 5; **D**, GK *n* = 9). Data are presented as mean ± SEM as percentage contraction of KCl. * *p* < 0.05; ** *p* < 0.01; *** *p* < 0.001, calculated with two-way ANOVA.

Since we observed an altered endothelial function in aortas and mesenteric arteries from T2D animals, we next applied a novel dinucleotide Up_4_A to stimulate PRs in aortas and mesenteric arteries from WT and GK rats. Up_4_A produced more potent contraction in mesenteric arteries than in aortas of WT rats (Figure 5). The vasoconstrictor response was markedly enhanced in aortas (Figure 5A), but not mesenteric arteries (Figure 5B) from GK as compared to WT rats. These observations again support that involvement of PRs is altered in aortas in T2D. Interestingly, NO synthase inhibition with *N*^(G)^-Nitro-*L*-arginine methyl ester (*L*-NAME) significantly enhanced Up_4_A-induced contraction in aortas in both WT and GK rats (Figure 5C,D). The effect of *L*-NAME on Up_4_A-induced contraction was similar in WT and GK groups (Δ area under the curve: 30 ± 8 in WT and 29 ± 6 in GK rats).

### 2.5. Effects of the Non-Specific P1R and P2R Antagonists on Up_4_A-Mediated Vascular Contraction in Aortas

**Figure 6:**
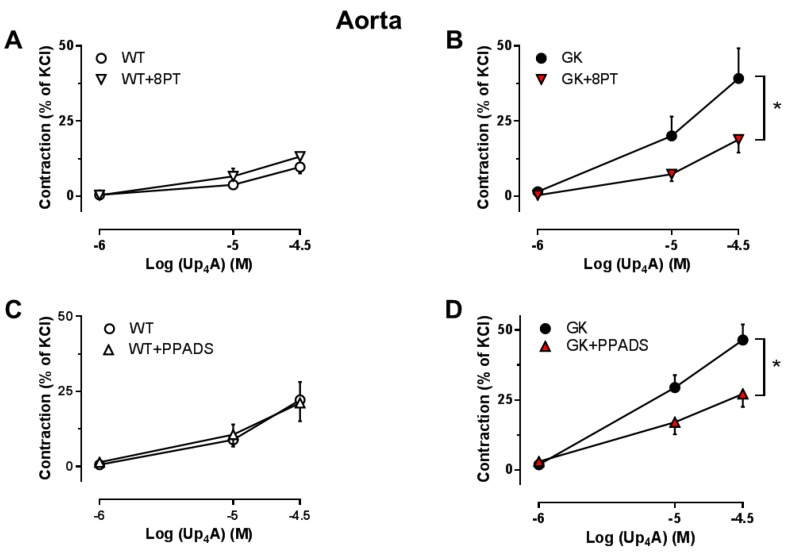
Effects of the P1R and P2R antagonists on vasoconstrictor response to Up_4_A in aortas from WT and GK rats. Concentration response curves for Up_4_A in aortas in the absence and presence of the non-selective P1R antagonist (8PT, 10^−5^ M) (**A**, WT *n* = 3; **B**, GK *n* = 8) and the non-selective P2R antagonist (PPADS, 10^−5^ M) (**C**, WT *n* = 4; **D**, GK *n* = 6). Data are presented as mean ± SEM as percentage contraction of KCl. * *p* < 0.05, calculated with two-way ANOVA.

Both the non-selective P1R antagonist 8PT and the non-selective P2R antagonist PPADS significantly attenuated the vasoconstrictor response to Up_4_A in aortas from GK rats (Figure 6B,D), but did not affect Up_4_A-mediated vascular contraction in aortas from WT rats (Figure 6A,C). This indicates that Up_4_A-enhanced aortic contraction in GK rats is attributable to activation of both P1Rs and P2Rs.

### 2.6. Effects of the Specific Antagonists for A1R, P2X_7_R and P2Y_6_R on Up_4_A-Mediated Vascular Contraction in Aortas

**Figure 7:**
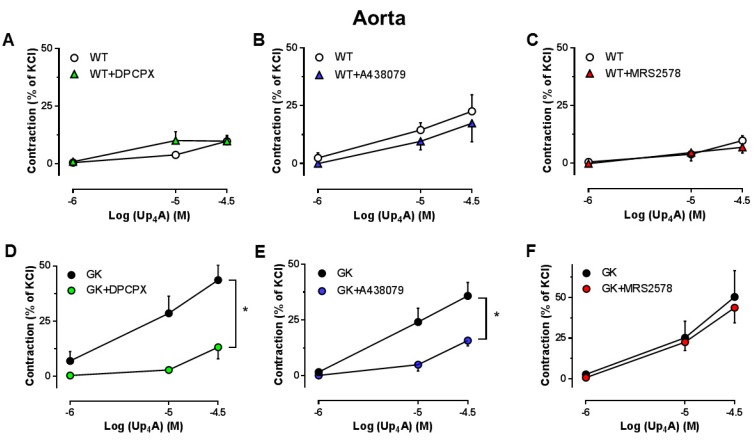
Effects of specific antagonism for A1R, P2X_7_R, and P2Y_6_R on vasoconstrictor response to Up_4_A in aortas from WT and GK rats. Concentration response curves for Up_4_A in aortas in the absence and presence of the A1R antagonist (DPCPX, 10^−8^ M) (**A**, WT *n* = 3; D, GK *n* = 4), the P2X_7_R antagonist (A438079, 10^−5^ M) (**B**, WT *n* = 6; E, GK *n* = 6), and the selective P2Y_6_R antagonist (MRS2578, 10^−5^ M) (**C**, WT *n* = 3; F, GK *n* = 4). Data are presented as mean ± SEM as percentage contraction of KCl. * *p* < 0.05 calculated with two-way ANOVA.

We next investigated the specific subtype(s) of PRs involved in the vasoconstrictor response to Up_4_A. A1R inhibition with DPCPX, P2X_7_R inhibition with A438079 but not P2Y_6_R inhibition with MRS2578 significantly attenuated the Up_4_A response in aortas from GK rats (Figure 7D–F). None of the antagonists affected the vasoconstrictor response to Up_4_A in aortas from WT rats (Figure 7A–C).

### 2.7. Protein Expression of A1R, P2X_7_R, and P2Y_6_R in Aortas from WT and GK Rats

**Figure 8:**
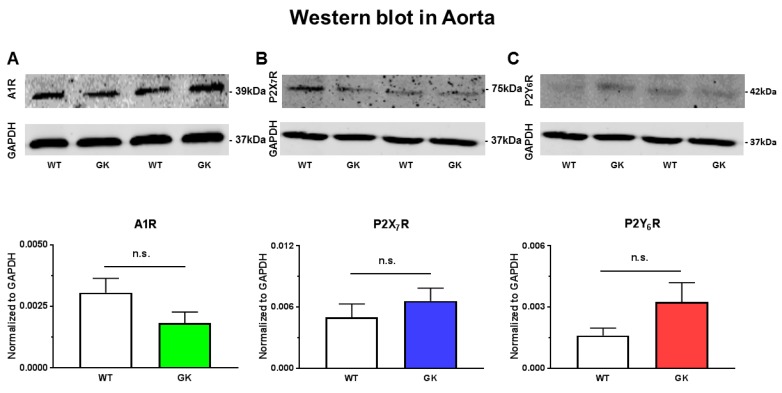
Protein expression of the A1R (**A**), P2X_7_R (**B**), and P2Y_6_R (**C**) in aortas from WT (*n* = 6) and GK rats (*n* = 8). Data are presented as mean ± SEM; n.s: not statistically significant with unpaired Student’s *t*-test.

An alteration in the expression of PRs in aortas could conceivably underlie the impaired endothelial function as well as the enhanced Up_4_A-mediated vasoconstrictor response in GK rats. We therefore determined the protein levels of A1R, P2X_7_R, and P2Y_6_R. No significant alterations in A1R (Figure 8A), P2X_7_R (Figure 8B), and P2Y_6_R (Figure 8C) were detected in aortas from GK rats as compared to WT rats.

## 3. Discussion

The main findings of the present study are that (1) endothelial dysfunction in aortas, but not mesenteric arteries of T2D rats was attenuated by the non-selective P1R and P2R antagonists; (2) the endothelial dysfunction in aortas was attributable to activation of A1R, P2X_7_R, and P2Y_6_R; (3) the vasoconstrictor responses to Up_4_A was enhanced in aortas, but not mesenteric arteries from T2D rats; (4) this enhancement in aortas was attenuated by the non-selective P1R and P2R antagonists as well as the specific antagonists for A1R and P2X_7_R, but was not affected by the P2Y_6_R antagonist; and (5) protein expression of A1R, P2X_7_R, and P2Y_6_R was unaltered in aortas from T2D rats. These results indicate that altered PR sensitivity is an important mechanism underlying endothelial dysfunction in aortas of T2D animals.

Endothelial dysfunction represents an early manifestation in vascular complications associated with diabetes and is well established in both patients and rodent models with T2D [20,21,22,23]. In accordance with previous studies, there is endothelial dysfunction in both conduit and resistance arteries in spontaneously developed T2D of non-obese GK rats [24,25].

Activation of PRs by various nucleot(s)ides plays a pivotal role in the control of vascular function [4]. Of note, endogenous nucleotides and nucleoside or their nucleotidases are altered in diabetes [26], which may affect the sensitivity of PRs on vasculature in response to pharmacological stimulations [7,8,9,10,27]. In the present study, PR sensitivity seems to be altered in conduit, but not resistance arteries, as evidenced by that non-selective inhibition for P1Rs and P2Rs with 8PT and PPADS at concentrations of 10 µM improved endothelial function in aortas, but not mesenteric arteries from GK rats. Similarly, contractions of aortas but not mesenteric arteries from GK rats were enhanced by Up_4_A which activates P1Rs, most P2XRs and P2YRs [8,15,28], and the enhancement in aortas by Up_4_A was attenuated by both P1R and P2R inhibition. This is supported by the observation that adenosine-mediated purinergic signaling is altered in aortas, but not mesenteric arteries of a diabetic mouse model [29]. In contrast, using purines as stimulators, both ATP (activates most of P2XRs and possibly P2Y_1_R) [30] and UTP (activates P2Y_2_R and P2Y_4_R) [30] enhanced contractions of mesenteric arteries of GK rats, an effect that was attenuated by non-selective P2R inhibition [11]. The discrepancy regarding the alteration of purinergic signaling in mesenteric arteries may be due to the age of animal used (15–18 weeks in the present study vs. 37-42 weeks in the study mentioned above). The altered purinergic activation likely appears when the vasoconstrictor response to purine stimulation e.g., ATP wanes in mesenteric arteries of older healthy rats but is maintained in aged GK rats [31]. Future studies are needed to confirm such explanations. The altered PR sensitivity in aortas from GK rats likely presents at endothelial level, as inhibition of NO, the major endothelial component, enhanced Up_4_A-induced contraction to a similar extent between WT and GK rats. Interestingly, the non-selective P2R antagonist PPADS at 10 µM significantly attenuated EDR in aortas from WT rats. Activation of P2X_1_R; P2X_2_R; and possibly also P2X_3_R, P2X_4_R P2X_7_R, P2Y_1_R, P2Y_2_R, P2Y_4_R, and P2Y_11_R on endothelial cells has been shown to produce NO and prostacyclin with subsequent vasodilation [32]. Given the ability of PPADS at concentration of 10 µM to inhibit most of P2XRs mentioned above [33], PPADS appeared to inhibit some of those vasodilator PRs resulting in an impaired EDR in aortas from WT rats [16]. These observations also indicate that there is likely a shift from the vasodilator P2Rs to vasoconstrictor P2Rs in diabetes.

We further investigated the involvement of PR subtypes in vascular function in aortas of T2D animals. A1R inhibition with DPCPX significantly improved endothelial function in GK rats. Using Up_4_A as stimulator, the increased contraction was attenuated by DPCPX in GK rats, despite an unaltered A1R expression. These findings suggest that A1R sensitivity is altered involving in endothelial dysfunction in T2D. A1R is expressed in the vasculature and activation of A1R typically results in vascular contraction [34]. The A1R-mediated contraction in aortas has been observed to be reduced in a diabetic mouse model without obvious endothelial dysfunction [29]. The decreased A1R activation is due to a compensatory mechanism to counterbalance the increased adrenergic vascular contraction [29]. In addition to A1R, activation of A3R typically produces vasoconstriction [34]. However, the evidence that the selective A3R agonist did not affect vascular function in aortas of mice with diabetes [29] and Up_4_A-mediated aortic contraction is not altered in A3R knock-out mice [16] suggests that A3R is unlikely to be involved in endothelial dysfunction in diabetes. Similarly, P2X_7_R inhibition with A438079 improved endothelial function and attenuated the Up_4_A-increased contraction in GK rats, despite an unaltered P2X_7_R expression. This suggests increased P2X_7_R sensitivity accounts for endothelial dysfunction in T2D. The importance of P2X_7_R among other P2XRs in regulation of vascular function in diabetes has been addressed in several studies. Thus, renal vascular reactivity is enhanced in response to ATP in diabetic rats through P2X_7_R [35]. Downregulation of P2X_7_R in coronary microcirculation of diabetic swine is compensated by enhancement of Up_4_A-mediated other vasodilator PRs [8]. Moreover, P2X_7_R activation accelerates retinal microvascular dysfunction in diabetic rabbits [36]. Activation of P2YRs generally results in vasodilation [32]. P2Y_6_R has been shown to be the predominant contractile receptor for both UTP and UDP-induced contraction in coronary vasculature with larger diameter, but acts as the vasodilator receptor in vessels with smaller diameter [37]. In the present study, P2Y_6_R inhibition with MRS2578 significantly improved endothelial function in GK rats, suggesting an involvement of P2Y_6_R in endothelial dysfunction in T2D. A similar upregulation of endothelial P2Y_6_R mediating increased UDP-induced relaxation in aortas of diabetic OLETF rats has been identified [38]. As the expression of P2Y_6_R did not significantly differ between GK and WT rats, increased P2Y_6_R sensitivity may exist in aortas of T2D accounting for the endothelial dysfunction. In contrast, P2Y_6_R inhibition had no effect on the Up_4_A-mediated aortic contraction in GK rats. Up_4_A has been shown to activate P2Y_6_R in coronary vasculature, where the P2Y_6_R-mediated relaxation is preserved in diabetic swine [8]. The finding that Up_4_A-mediated aortic contraction was not affected by MRS2578 in GK rats is likely due to the fact that Up_4_A does not activate P2Y_6_R in aortas, as Up_4_A does not significantly affect P2Y_6_R expression in aortic endothelial cells [39]. Altered PR sensitivity but not expression in diabetes is commonly observed in previous studies. Thus, activation of P2Y_1_R contributes to impaired relaxation in mesenteric arteries and activation of P2Y_2_R and P2Y_4_R accounts for the increased ATP-induced contraction in mesenteric arteries in diabetes without significant changes in receptor protein expression [11,12]. Moreover, as key purinergic receptors mediating Up_4_A-enhanced contraction, P2X_1_R and P2Y_2_R protein expression do not differ in renal arteries of WT and GK rats [10]. This is also supported by our previous findings that the sensitivity but not mRNA level of P2Y_1_R is increased to Up_4_A in coronary vasculature of diabetes [8].

Existing evidence indicate that endogenous ROS formation is greater in aortas from GK rats as compared to WT rats [40]. We further demonstrated that ROS inhibition improves endothelial function in aortas from GK rats [21]. Of importance, activation of P2X_7_R results in ROS production in endothelial cells with high glucose stimulation [41]. Up_4_A-induced contraction in aortas from WT rats is through P1R and ROS [18]. In addition to ROS, Up_4_A activates P2R to generate TxA2 leading to vascular contraction in mouse aortas [16]. Of note, Up_4_A-mediated coronary relaxation in diabetes is reduced via increased TxA2 [8]. Up_4_A-enhanced renal contraction in GK rats is via increased TxA2 receptor sensitivity [10]. Taken together, these findings imply that generation of ROS and TxA2 may also result from altered PR sensitivity induced by Up_4_A as potential post-receptor mechanisms in GK aortas leading to vascular dysfunction. Interestingly, an interaction between A1R and P2X_7_R exists [42]. The speculation that A1R interacts with P2X_7_R resulting in ROS production in our model may explain the similar inhibitory effect of the A1R or P2X_7_R antagonist on Up_4_A-induced contraction in aortas from GK rats. Further investigations are needed to elucidate exact mechanisms.

Some limitations of the present study need to be acknowledged. All experiments and pharmacological agents were applied ex vivo, which does not reflect the in vivo situation fully. Future studies are needed to validate endothelial function in vivo and this will definitely give more insights on the purinergic regulation of vascular function in diabetes. In addition, the lack of selective antagonists targeting several subtypes of PRs limits the delineation of the involvement of other PRs in the regulation of vascular function in T2D.

In conclusion, our findings indicate that the sensitivity of PRs are altered in T2D conduit arteries. Inhibition of several PRs individually results not only in improvement in EDR, but also in attenuation of the vasoconstrictor response to Up_4_A in T2D. These results imply that targeting PRs could serve as potential therapy for improving vascular function in patients with T2D. 

## 4. Materials and Methods

### 4.1. Animals

All experimental protocols were performed in accordance with the Guide for Care and Use of Laboratory Animals (NIH publication no. 85–23, revised 1996) and approved by the regional ethical committee for animal experiments in Stockholm (ethical number: N108/14; approval date: 22 May 2014).

A total of 21 healthy male WT rats (14–18 weeks old on the day of experiment) and 37 age and sex-matched GK rats that spontaneously developed T2D were used in the present study. WT rats were purchased from Charles River (Sulzfeld, Germany) and housed in the animal facility of Karolinska University Hospital (L5). GK rats were derived from glucose intolerant WT rats and were bred in the animal facility of the Department of Molecular Medicine and Surgery, Karolinska Institute. The GK strain was established from normoglycemic WT rats by repeated inbreeding in each successive generation of the siblings with the highest blood glucose levels during an oral glucose tolerance test [43]. All animals were kept at 22 °C with 12 h light/dark cycle with free access to standard chow and water.

### 4.2. Tissue Preparations and Wire Myograph Protocols

Rats were anesthetized with pentobarbital sodium (50 mg/kg i.p) followed by thoracotomy and removal of aortas and guts. The rat aortas and mesenteric arteries were dissected, placed into Krebs–Henseleit (KH) buffer on ice and cleaned by removing fat and connective tissues under microscope, and subsequently cut transversely into 2 mm rings. Arterial segments were either used immediately for functional experiments or stored in −80 °C for later expression analysis. The aortic rings and mesenteric arteries were mounted in wire myograph (Danish Myo Technology, Aarhus, Denmark) in separate 6 ml organ baths containing KH buffer. The KH buffer (pH 7.4) containing (in mM) 118 NaCl, 4.7 KCl, 1.2 MgSO_4_, 1.2 KH_2_PO_2_, 25 NaHCO_3_, 11 glucose, and 2.4 CaCl_2_ was maintained at 37 °C and aerated with 95% O_2_/5% CO_2_. Changes in contractile forces were recorded with a Harvard isometric transducer. Following a 30 min stabilization period, the internal diameter was set at a tension equivalent to 0.9 times the estimated diameter at 100 mmHg effective transmural pressure [6,21]. At the end of the equilibration period, the vessels were exposed to KCl twice (50 mM and 100 mM, respectively for aortas; 50 mM each time for mesenteric arteries) to check the contractility. Thereafter, vessels were allowed to equilibrate in fresh KH buffer for 30 min before initiating different experimental protocols. For determination of PR involvement, the non-selective P1R antagonist 8PT (10 µM) [8,44], the non-selective P2R antagonist PPADS (10 µM) [8,45], the A1R antagonist DPCPX (10 nM) [16,46], the P2X_7_R antagonist A438079 (10 µM) [8,47], and the P2Y_6_R antagonist MRS2578 (10 µM) [8,38,39] were added in the organ bath 30 min before pre-constriction with 10^−6^ M phenylephrine (PE). EDR and EIR were determined by administration of increasing concentrations (10^−9^–10^−5^ µM) of ACh and SNP, respectively [21]. In separate experiments, Up_4_A contraction responses (10^−6^–3 × 10^−5^ µM) were conducted in vessel segments without preconstriction in the absence and presence of the purinergic antagonists mentioned above as well as the NO synthase inhibitor *L*-NAME (100 µM) [17].

### 4.3. Western Blotting

Rat aortas were lyzed with RIPA lysis buffer (Amresco, Solon, OH, USA) containing protease inhibitors (Roche, Mannheim, Germany) with subsequent homogenization and centrifugation at 12,000 *g* at 4 °C for 20 min. Protein content was quantified with bicinchoninic acid protein assay kit (Pierce Biotechnology, Life Technologies). The proteins were separated on 10% SDS gel (30 μg per sample) and transferred onto 0.45 μm nitrocellulose blotting membranes (Amersham, Freiburg, Germany). Membranes were blocked with 5% milk for 1 h at room temperature and incubated overnight at 4 °C with primary antibodies against A1R (1:500, Sigma-Aldrich, SL, USA, product number: A-268), P2X_7_R (1:200, Alomone Labs, Israel, product number: APR-004), P2Y_6_R (1:200, Alomone Labs, Israel, product number: APR-011) and GAPDH (1:2500, Sigma-Aldrich, MO, USA, product number: G9545), followed by incubation with secondary antibodies for 1 h at room temperature (goat anti-rabbit, 1:20,000, LICOR IR Dye 800CW, product number: 926-32211) [21]. Band densities were analyzed with Image Studio Lite Version 5.2 (LI-COR Bio-sciences, Bad Homburg, Germany). Data obtained were normalized to GAPDH and displayed with arbitrary units.

### 4.4. Statistical Analysis 

Vascular relaxation to ACh or SNP was expressed as percentage of contraction to PE. Vascular contraction responses to Up_4_A were expressed as percentage of contraction to the second exposure to KCl. The effect of drug treatment on the relaxation and contraction responses were assessed using two-way analysis of variance for repeated measures. Unpaired *t*-test was used for comparison between two groups. n refers to the number of animals each analysis was made upon. All data are represented as means ± SEM. All the statistical analysis in the present study were performed with Prism 7.0, GraphPad, San Diego, CA, USA. Two-sided *p* < 0.05 was considered as statistically significant.

## Abbreviations

Ach	acetylcholine
DPCPX	dipropylcyclopentylxanthine
EDR	endothelium-dependent relaxation
EIDR	endothelium-independent relaxation
GK	Goto Kakizaki
*L*-NAME	*N*^(G)^-Nitro-*L*-arginine methyl ester
OLETF	Otsuka Long-Evans Tokushima fatty
PE	phenylephrine
PPADS	pyridoxal phosphate-6-azo(benzene-2,4-disulfonic acid)
PR	purinergic receptor
ROS	reactive oxygen species
SNP	sodium nitroprusside
T2D	type 2 diabetes
Up_4_A	uridine adenosine tetraphosphate
WT	Wistar
